# Exploring the Impact of the COVID-19 Pandemic on Learning Experience, Mental Health, Adaptability, and Resilience Among Health Informatics Master’s Students: Focus Group Study

**DOI:** 10.2196/63708

**Published:** 2025-02-10

**Authors:** Nadia Davoody, Natalia Stathakarou, Cara Swain, Stefano Bonacina

**Affiliations:** 1 Health Informatics Centre, Department of Learning, Informatics, Management and Ethics Karolinska Institutet Stockholm Sweden; 2 Academic Department of Military Surgery & Trauma Royal Centre for Defence Medicine Birmingham United Kingdom

**Keywords:** COVID-19 pandemic, eHealth, blended learning, health informatics, higher education adaptation

## Abstract

**Background:**

The shift to online education due to the COVID-19 pandemic posed significant challenges and opportunities for students, affecting their academic performance, mental well-being, and engagement.

**Objective:**

This study aimed to explore the overall learning experience among health informatics master’s students at Karolinska Institutet, Sweden, and the strategies they used to overcome learning challenges posed by the COVID-19 pandemic.

**Methods:**

Through 3 structured focus groups, this study explored health informatics master’s students’ experiences of shifting learning environments for classes that started in 2019, 2020, and 2021. All focus group sessions were recorded and transcribed verbatim. Inductive content analysis was used to analyze the data.

**Results:**

The results highlight the benefits of increased autonomy and flexibility and identify challenges such as technical difficulties, diminished social interactions, and psychological impacts. This study underscores the importance of effective online educational strategies, technological preparedness, and support systems to enhance student learning experiences during emergencies. The findings of this study highlight implications for educators, students, and higher education institutions to embrace adaptation and foster innovation. Implications for educators, students, and higher education institutions include the need for educators to stay current with the latest educational technologies and design teaching strategies and pedagogical approaches suited to both online and in-person settings to effectively foster student engagement. Students must be informed about the technological requirements for online learning and adequately prepared to meet them. Institutions play a critical role in ensuring equitable access to technology, guiding and supporting educators in adopting innovative tools and methods, and offering mental health resources to assist students in overcoming the challenges of evolving educational environments.

**Conclusions:**

This research contributes to understanding the complexities of transitioning to online learning in urgent circumstances and offers insights for better preparing educational institutions for future pandemics.

## Introduction

### Background

The COVID-19 pandemic disrupted education worldwide, and many universities were required to shift to online education despite most being unprepared for such a shift [1]. The education response during the early phase of the COVID-19 pandemic focused on implementing remote learning modalities as an emergency response and mostly on the online delivery of educational material. The result of these efforts was a substantive rise in e-learning, whereby teaching and learning activities took place remotely via digital platforms. After the pandemic, the use of e-learning was expected to grow [1].

e-Learning is the use of internet technologies to enhance knowledge and performance. e-Learning technologies offer learners control over the content, learning sequence, pace of learning, time, and often media, allowing them to tailor their experiences to meet their learning objectives [2]. Within the context of the COVID-19 pandemic, e-learning and online learning refer to remote teaching strategies and methods that universities used to urgently respond to the requirements of health protocols and restrictions in mobility [3].

While e-learning holds significant potential, the rapid deployment of strategies during the pandemic showed mixed results. Most of the swift adoption focused on reaching all students and enhancing accessibility, but educators lacked the time to develop and implement pedagogical strategies that could enhance the learning experience [3]. Strategies used for short-term online education may not have been suitable for the prolonged disruption caused by the pandemic.

Student experiences with e-learning during the pandemic were mixed. Some studies reported a “new normal” that students viewed positively, citing benefits such as education continuity, increased accessibility, stronger learner-lecturer interactions, and greater confidence in expressing themselves in an online learning environment [1,4-7].

Within the field of medical education, one study reported that students responded positively to the transition to online learning methods, with a notable improvement in student satisfaction related to course structure [8]. Another study highlighted medical students’ generally positive attitudes toward e-learning during the pandemic. However, while e-learning was seen as a necessary and beneficial alternative, challenges were recognized, such as increased stress and anxiety, limited internet access, technical difficulties, and reduced hands-on clinical training [9]. Health profession education programs have reported issues in transitioning to online learning and maintaining continuity in education [10]. There have been difficulties related to adapting traditional teaching methods to online formats and the stress this placed on both students and educators [11]. Stress, unfamiliarity with online classrooms, uncertainty about academic futures, and the rapid shift to e-learning contributed to negative experiences for many students [12,13]. In addition, insufficient training, inadequate internet infrastructure in some countries, and lack of preparation led to poor student experiences, undermining the sustainability of e-learning [14].

The contradictory evidence on diverse experiences of e-learning during the pandemic underscores the need for further investigation within this area. To better prepare for future crises, higher education institutions need to understand the experiences of both educators and students and develop educational strategies for online learning during emergencies. Although many studies have investigated students’ learning experiences during the pandemic, there are limited insights into the specific challenges and strategies used to overcome them. Contextual factors such as internet access, cultural differences, and subject of study likely influenced the experiences and perceptions of both students and educators. Therefore, there is a need to further explore e-learning experiences and strategies across different contexts and subjects of study. Health informatics education uniquely integrates theoretical knowledge with applied technical and health care–related skills, which may have been significantly affected by the shift to online learning.

### Study Aim and Research Question

This study aimed to explore the overall learning experience of health informatics master’s students at Karolinska Institutet (KI), Sweden, and the strategies they used to overcome the learning challenges posed by the COVID-19 pandemic. This study addressed the following research question: How did health informatics master’s students at KI experience learning during the COVID-19 pandemic, and what strategies did they use to overcome related challenges?

## Methods

### Study Design

In this study, we conducted 3 semistructured focus group interviews comprising a total of 16 registered students and alumni of the Master’s Programme in Health Informatics. The COREQ (Consolidated Criteria for Reporting Qualitative Research) guidelines [15] were used for reporting the results. The process was piloted, and questions for the participants were determined in advance by authors ND, SB, and NS adapted from previous studies on the impact of the COVID-19 pandemic on medical education [9] and on teaching and learning in health professional education [11].

In this explorative study, focus group interviews were chosen as a data collection technique as they allow for the generation of rich qualitative data through group interactions and dynamics, which is beneficial when addressing an exploratory question. Participants can build on each other’s ideas, leading to more nuanced insights [16,17]. During these focus groups, the students could share their experiences and perspectives as part of an open-ended discussion, leading to a deeper understanding of their learning experiences and the strategies used to overcome challenges during the COVID-19 pandemic.

Given the exploratory study design, focus groups are ideal as a data collection method as they facilitate open-ended discussion and are effective in collecting rich data that might not have been possible to collect through more structured data collection methods. The focus groups were held remotely using the Zoom platform (Zoom Video Communications) with CS in the role of interviewer.

### Study Setting and Participants

The context of the study was the Master’s Programme in Health Informatics provided by the Department of Learning, Informatics, Management, and Ethics at KI. It is a 2-year global program run jointly with Stockholm University. The program is designed for students with an interest in IT and how it can be applied to the fields of medicine and health care. As such, the students may have either a technical or health care background.

Convenience sampling was used to recruit participants. Individuals who had participated in the master’s program during the COVID-19 pandemic were invited to contribute. Therefore, all participants were alumni or active registered students split into 3 cohorts: students who had registered in 2019 and graduated in 2021 (cohort 1), students who started in 2020 and graduated in 2022 (cohort 2), and students who started in 2021 and graduated in 2023 (cohort 3). Table 1 provides some characteristics of the participants. Most participants (13/16, 81%) were living in Sweden; however, as some of the students had relocated to their native countries or were working overseas, this informed the decision to conduct online focus groups.

**Figure 1:**
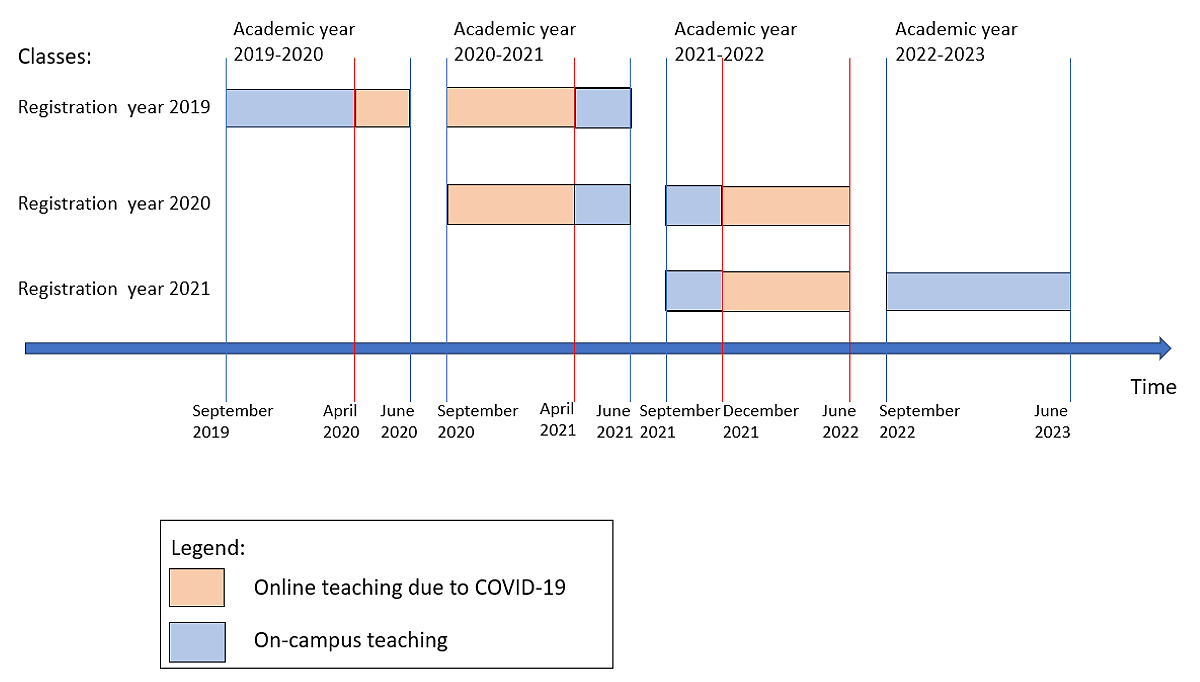
On-campus and online teaching during the COVID-19 pandemic.

Figure 1 shows the timeline of how the Master’s Programme in Health Informatics from the academic year 2019-2020 to 2022-2023 was conducted. On-campus teaching and online teaching due to the COVID-19 pandemic are indicated using distinct colors. Students that started the program in September 2020 mostly received online teaching.

**Table 1:** Participant characteristics.

	Background	Experience with online learning
2019_Participant 1	Health care	No
2019_Participant 2	Health care	Yes
2019_Participant 3	Health care	No
2020_Participant 1	IT or technical	No
2020_Participant 2	Health care	Yes
2020_Participant 3	Health care	Yes
2020_Participant 4	Health care	Yes
2020_Participant 5	Health care	Yes
2020_Participant 6	Health care	No
2020_Participant 7	IT or technical	No
2020_Participant 8	Health care	Yes
2021_Participant 1	IT or technical	Yes
2021_Participant 2	IT or technical	Yes
2021_Participant 3	Health care	Yes
2021_Participant 4	Health care	Yes
2021_Participant 5	Health care	Yes

### Data Collection and Analysis

A total of 3 online focus group interviews were conducted during 2023 using the Zoom platform; the interviews were recorded and transcribed verbatim. To reduce familiarity bias in the study, CS, who had not been involved in teaching within the health informatics program, conducted the focus groups. The first focus group included 3 students from cohort 1, the second focus group included 8 students from cohort 2, and the third and final focus group included 5 students from cohort 3. The first focus group had a duration of 45 minutes, with the other 2 focus groups lasting approximately 1 hour and 30 minutes. The piloting process did not generate any data and was not included in the analysis.

Generated data were analyzed using inductive content analysis [18]. The inductive content analysis allowed for themes and patterns to be constructed directly from the data that were grounded in the students’ actual experiences rather than imposing preconceived categories. A combination of coding methods was used: descriptive coding summarizes the main topics of the text, and pattern coding was used to condense meaning units into broader patterns to group the initial codes into broader themes. Pattern coding helps identify and understand the broader patterns and relationships within the data [19]. ND, NS, and SB conducted the initial coding. Each coder was instructed to familiarize themselves with the data, read through the entire dataset to gain an overall understanding before starting the coding process, identify meaning units, condense them, and assign codes that captured the essence of each unit. They independently reviewed all transcribed interviews, dividing the responsibilities for identifying relevant meaning units and conducting the initial coding. CS joined the analytical process once initial coding and subcategories had been generated. Upon identifying subcategories, a comparative analysis was conducted to reveal similarities and differences among student groups. Comparative analysis was conducted through peer debriefing sessions in which ND, NS, and SB compared their assigned codes and discussed differences in interpretations. All authors reviewed discrepancies collaboratively until a consensus was reached. Discrepancies were resolved by re-examining the raw data for the meaning unit in question, discussing interpretations considering the research aim, and refining the coding if needed. At this point, it was decided to collaboratively proceed with the categorization and identification of subthemes for all 3 student groups. This not only minimized the recurrence of redundant findings resulting from similarities among groups but also empowered us to emphasize the subcategories in which different student groups expressed distinct experiences or opinions.

### Ethical Considerations

This research was carried out in Sweden. According to the Swedish Ethical Review Act, the research presented in our submitted manuscript did not require ethics approval as it did not handle sensitive personal information (as understood by the European General Data Protection Regulation). However, ethical requirements still apply, and written informed consent to take part in the study was obtained from all participants. The consent form outlined the study’s purpose, potential risks and discomforts, the voluntary nature of participation, and the right to withdraw at any time. It also stated that no compensation would be provided for participation. Participants were assured that their confidentiality and privacy would be preserved.

## Results

### Overview of Data Collection and Data Analysis

Our analysis of the generated focus group data identified 1 main theme—*adapting to hybrid learning*—and three subthemes: (1) students’ considerations of learning during the pandemic, (2) moving between learning environments, and (3) students’ well-being and engagement in learning. Each subtheme included several categories relevant to all student groups, summarized in Table 2.

The overarching theme of *adapting to hybrid learning* highlights the challenges and experiences faced by participants navigating the transition from traditional on-campus education to online learning and vice versa. It includes the autonomy that students seek in shaping their learning experiences, the integration of learning into their daily lives, and the varying perceptions of online education as an obligation rather than a choice during the COVID-19 pandemic. It further explores the changed aspects of social interaction, the struggle with engagement in online learning, the technical challenges faced, and the diverse technological readiness levels for different learning environments. In addition, this theme addresses the psychological impact of remote learning and the need for adequate support, the varied levels of student motivation, the influence of family and pet support, and the observed lack of networking opportunities and social interaction in online educational settings.

**Table 2:** An overview of the subcategories, categories, subthemes, and themes.

Theme, subtheme, and category				Subcategories
**Adapting to hybrid learning**				
	**Students’ considerations of learning during the pandemic**			
		Benefits	Autonomy over the learning experience Integrating learning into their lives	
		Challenges	Altered social interaction Technical challenges	
	**Moving between learning environments**			
		Transition—campus to online education	Online education as an obligation, not an option Technological readiness for different learning environments Psychological impact and support	
		Transition—online to campus education	Technological readiness for different learning environments Lack of engagement in online learning Different experiences in interaction with classmates and teachers between online and campus education	
	**Students’ well-being and engagement in learning**			
		Motivation level Strategies to stay motivated	Varied levels of motivation Group work as a connecting element Family and pet support	
		The impact of online learning on academic performance	Autonomy Lack of networking (social interaction)	
		Overall experience and recommendations	Improving online sessions Improving educational support and delivery	

### Students’ Considerations of Learning During the Pandemic

**Figure 2:**
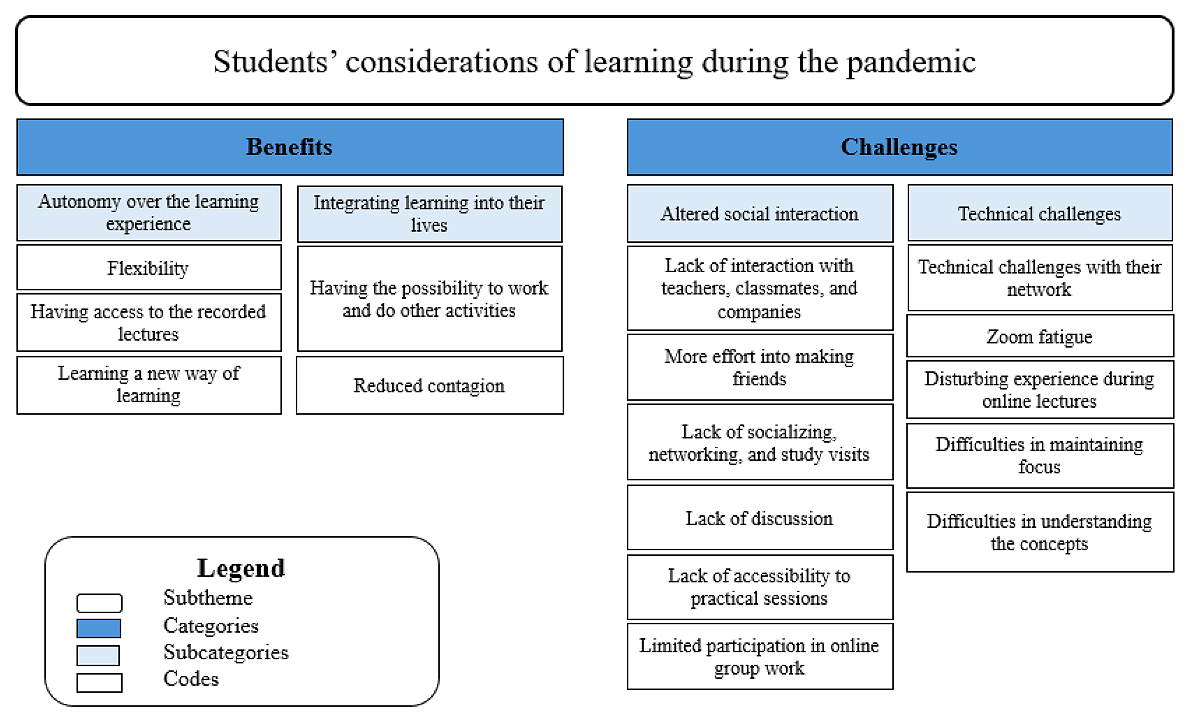
A hierarchical structure displaying the subtheme and codes for theme 1—students’ considerations related to learning during the pandemic.

The students recognized that the changes and requirements necessitated by the COVID-19 pandemic brought both benefits and challenges. Figure 2 illustrates the relationship among the codes, subcategories, and categories for the subtheme *students’ consideration of learning during the pandemic*.

#### Benefits

Benefits were characterized by increased *autonomy over their learning experience* and an ability to *integrate learning more readily into their lives*. Most of the participants mentioned that *flexibility* was one of the biggest benefits during the COVID-19 pandemic. Some mentioned that they could work from home or, in the case of many international students attending the Master’s Programme in Health Informatics at KI, even work in their home country:

[The] main benefit is that it was less time-consuming. I was living at that time in [area_1] and I would have to travel to [area_2], that time was saved for me.

2019_Participant 3

I can start with one of the big benefits I had, especially in the beginning was that I was able to start the master’s program from Germany where I am originally coming from. So, I moved I think 2 weeks in the program to Sweden. So, that was a big benefit to be more flexible in terms of the location for sure.

2020_Participant 1

*Having access to the recorded lectures* was appreciated as it made it possible for the students to go through the material whenever it was suitable for them. In addition, some of the students believed that they had *learned a new way of learning* and could now learn anything online. Online learning was believed to be more *efficient* as they could save time without the requirement to commute to campus:

...For me, it helped me to have the independence to learn as [participant] said. And yeah, and like now I can I have the feeling that I can learn anything online, I can just sign up for a course and in some videos and do it. And yeah, and in my current job, I did this a lot during the last six months. New technologies, new programming languages, everything...

2020_Participant 7

...I do watch tutorials on YouTube right now, but you get used to it. To manage to solve problems alone actually, which is really interesting because it’s something that I do in my current job. If I don’t know how to do something, I watch YouTube. I’m not going to ask anybody, and I think that’s something with the pandemic as well...I managed to solve the problems myself.

2020_Participant 4

*Having the possibility to work and do other activities* in parallel with their studies was also appreciated by some of the participants:

In terms of benefits, it was very beneficial to save the commute time to the university by at least two hours a day. I was able to work in parallel with my studies. That wasn’t a big deal. The third benefit, I would say [was] the flexibility to schedule meetings with colleagues in Group work. It’s much easier than scheduling a physical meeting...

2020_Participant 7

As the risk of being infected and getting sick was high during the COVID-19 pandemic, the *reduced contagion* was also perceived as a benefit by many students as they lived with their families and wished to protect or shield them or, at least, lower the transmission risk:

Yes, of course. But before that, I would like to add one thing that I think it hasn’t been mentioned by my fellow friend is that the advantages of online class or online learning during the pandemic is that we were able to refrain from the infection for COVID cases, of course, and I do believe that in Sweden it’s well managed about the cases or the infection rate is remain low, but you know the preventive measures that we stay in our home and limiting interaction that’s is also one.

2021_Participant 5

#### Challenges

The challenges that students experienced were divided into 2 distinct categories: *altered social interaction* and *technical challenges*. Most of the participants perceived the *lack of interaction with teachers, classmates, and companies* as a significant challenge of online learning. They experienced that the *group feeling* and the feeling of belonging to a bigger group was missing. Some students mentioned that they needed to put *more effort into making friends* and developing collegial relationships with their classmates. Several participants mentioned the challenges with the lack of *socializing, networking, and study visits* through online learning. This was noted through the *lack of discussion* due to people being shy and not turning on their cameras and by students leaving the online lectures directly after they finished:

...In this case, it’s an international program. And I was not living there, so I moved to this country to learn. But also like to meet new people, make friends, to expand my network. The interaction with the teachers. And as yeah, [participant] said to others you just attend the meeting, and then you close it, and you don’t have this interaction discussion afterward or during the class. So that was also a point, and I also like making friends. Of course, I made a lot of friends in this program. But you like the effort was bigger, you know? So, you have to be proactive to let’s meet. But in an in-person or personal program, you have more facilities...

2020_Participant 5

...another aspect that at least made me feel a bit I shouldn’t say depressed, but not as happy as I was not meeting all the people in the class. Partly built on that, you should form a team over two years, and that was for me very, very obvious that I almost missed that team spirit or the team.

2021_Participant 1

*Lack of accessibility to practical sessions* on campus and *limited participation in online group work* that might have resulted in the low quality of group work were other challenges mentioned by the students:

...I don’t have technical problems with attending online lectures...the only thing, as I mentioned before, is we had no opportunity to have someone...to have an instructor while we were doing these programming things...having online lectures is fine but having a technical lab doesn’t always work online.

2019_Participant 2

...regarding the challenge of collaboration...we had like several group assignments where we needed to have a lot of discussions...that was fairly difficult because we tend to have passive collaboration, I mean like normally one or two people lead while the others just agree it’s totally different from what we have in the class where everyone can jump in and share their talks during the group work.

2021_Participant 1

*Technical challenges* associated with online learning were discussed by students of all cohorts. Some students mentioned that they were worried about the reliability of their network along with some *technical challenges with their network*. For some students, it was difficult to work with Zoom in the beginning. There was also an adjustment period required to use online platforms for longer periods, such as experiencing *Zoom fatigue* from having several hours of online classes. There were additional technical problems with hybrid sessions, and some of the participants mentioned the *disturbing experience* that they had during online learning as some people spoke at the same time, which disrupted the flow of the conversation. During hybrid learning sessions, there was a lack of interaction between those online and those present in the room on campus:

So, in the beginning, I mean [during] the lectures sometimes there were some network issues, and then when you are the first time sitting on Zoom, and everybody has their cameras turned off.

2019_Participant 2

...Zoom fatigue because I think during that period of time, we had two or three classes in one day. Even with a normal session like from 9:00 to 3:00, we felt really exhausted and it was worsened when we had the online classes, and I was thinking that perhaps some sessions could be minimized. I mean like normally we will like 90 minutes for a single theoretical class. But if online sessions can be reduced to about 30 or 40 minutes and the rest of the time, we could do our self-learning. I think that would have been more beneficial.

2021_Participant 1

...One negative aspect I noticed was that I didn’t feel comfortable interrupting to ask a question. I know I would get distracted because it’s an online setting, and sometimes when two people speak at the same time, it can be very disruptive...also there are always some technical difficulties.

2021_Participant 2

While some students found online learning beneficial as it allowed them to concentrate better during lectures by multitasking and engaging in other activities simultaneously, others reported *difficulties in maintaining focus*. These students struggled with reduced attention spans, often due to the distractions of being at home with their families or lack of camera use during online lectures, which contributed to a sense of disengagement:

...Then the other thing is that I cannot focus properly because my kids are small. When I was at home, it was distracting many, many times and difficult...

2019_Participant 3

As a result, some students experienced *difficulties in understanding the concepts* and needed some assistance:

...Well, I think the plus point was that my husband works in technology, so I could take his help to understand the concepts and I think that was the main part that kept me going because if I don’t understand things, I just...I can’t focus and I can’t move forward...

2019_Participant 2

### Moving Between Learning Environments

**Figure 3:**
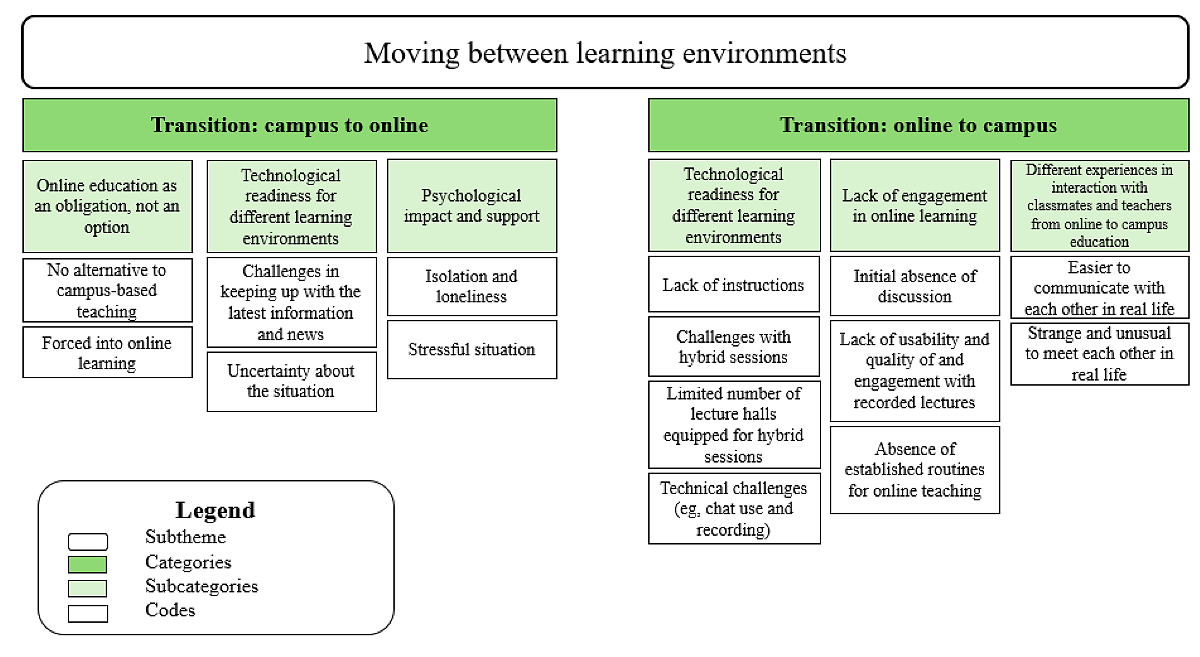
A hierarchical structure displaying the subtheme and codes for theme 2—moving between learning environments.

Figure 3 illustrates the relationship among the codes, subcategories, and categories for the subtheme *moving between learning environments*.

#### Transition: Campus to Online Education

The COVID-19 restrictions prompted universities to shift from on-campus to online teaching. The disease spread rapidly, rendering on-campus lectures unfeasible. Numerous international students faced *challenges in keeping up with the latest information and news*. Despite the continuous updates available on the university’s website, many international students experienced *uncertainty about the situation* and when everything would go back to normal:

...and I think that the transition between these different ways of working, I think that was hard for all of us because, well, especially for me, since I was studying before. So, I got used to online learning and then I went back to traditional learning and was very excited about that. But then it went back to online learning again and I was like, huh, OK...

2021_Participant 2

Working *online was an obligation rather than an option* for students, which may have made it easier to accept and manage, at least in the early stages of the pandemic. Having *no alternative to campus-based teaching* and *being forced into it* made online learning an obligation rather than a choice:

Well, I mean, I think there was no other choice. Everything was shut down, and there was no question of going back to campus. Nobody knew when things would reopen again, so in the beginning, we were forced into it, left with no other option. But then, I think the transition was OK.

2019_Participant 1

However, the psychological impact and requirement of support were important when considering the transition from on-campus to online environments. *Isolation and loneliness* and *a stressful situation* were common themes among students who did not have significant social support, such as family members living in Sweden. Most of the international students leaving their home countries to study the program in Sweden experienced that they were isolated and felt lonely during the COVID-19 pandemic:

...Then we changed our routines, but always we had some fear because of the unknown and what would happen next. It was always stressful, and we didn’t meet our friends and teachers because from my side I don’t have any relatives here, just only people from the university then I felt isolation and loneliness, and something, something alwayswas in the back of my mind and a little difficult to focus on my studies...

2019_Participant 3

#### Transition: Online to Campus Education

*Technological readiness for different learning environments* was a subcategory of transitioning from on-campus to an online environment and on the return to on-campus learning. At the onset of the pandemic, some students encountered challenges with Zoom due to a *lack of instructions*, particularly affecting those without a technical background. Numerous students encountered issues with audio quality during lectures and faced *challenges with hybrid sessions*. The university was not fully prepared for online or hybrid formats due to the *limited number of lecture halls equipped for hybrid sessions*:

...I was a nurse, so I wasn’t used to using it [Zoom]...No, it was difficult in the beginning to get used to using Zoom and the online campus. Nobody was explaining to you how to use it in person, so you have to do tutorials online for everything you do...

2020_Participant 4

We would have that many problems in the classroom technology-wise, like not being able to record the meetings, for instance. That was a big loss. Not being able to join functionally from home because it was not great for you. You didn’t actually know what was happening in the classroom, so it was. I just wasn’t expecting that. The classrooms weren’t equipped to handle hybrid learning. If that was presented as an option, so.

2020_Participant 8

Some participants noted the *initial absence of discussion* on enhancing online learning at the onset of the COVID-19 pandemic. Nevertheless, they observed an increase in discussions on how online learning worked and how it could be improved as the pandemic progressed. In addition, at the beginning of the pandemic, technical challenges (eg, Zoom difficulties, uncertainties about chat use, and initial problems with recordings) were encountered. As the pandemic continued, these issues were addressed, leading to a gradual improvement in the online learning experience over time:

So, I think by the time we started [the online lectures] everything got improved especially when they started to record the lectures. Because I remember at the beginning the lecture was not recorded. But later lectures were recorded, and this was good.

2019_Participant 2

I mean for the teachers also, in the beginning, it was difficult...now if you see Zoom meetings are usually facilitated by one person who keeps an eye on the chat while the teacher is teaching. And then in between they ask questions. I mean it took a few months...and during the third semester, it became better towards the end of it. So, I think yes, I mean everybody adapted to it because that was the only way left.

2019_Participant 1

Other *technical challenges* included the requirement for extra tools such as headsets and screens, issues with hybrid teaching, and inadequate online content delivery, marked by a *lack of usability and quality of and engagement with recorded lectures* and an *absence of established routines for online teaching*:

...but that isn’t very equitable for those who might not have had headphones or something like that, or maybe lived in a very small apartment, even though I live in a small apartment. Well, then maybe have a family and other people at home, maybe they didn’t have any headphones, so maybe that’s an aspect of the required equipment for the most optimal way of learning remotely...

2021_Participant 2

...if you maybe go and look at a lecture or recorded video on YouTube. They are very different, and they are a lot more engaged. Even though they’re not live, so I do think that there should be some sort of, yeah, look over, like how you’re supposed to have an online lecture to maximize learning. Because, yeah, I’ve, I found it quite strange that the prerecorded ones were better than the live ones.

2021_Participant 4

Some students encountered challenges with active participation in online and hybrid sessions, which they experienced as a lack of engagement in online learning from learners and, at times, educators. They emphasized the need for implementing strategies to enhance engagement during online sessions:

It lies on the lecturers. I mean, they’re supposed to engage the students, and I understand from their perspective that it’s so hard to engage people on a computer, because the students usually don’t have their cameras on, usually, maybe even sit in their beds, listening to the lecture. And there are some lecturers who really, really try to be like: please turn on your cameras...I think that it requires maybe some standard routine for how we’re supposed to do remote learning, and I mean, just like any technical product that goes into implementation, we need to have change management afterward. We need to maybe have some implementation consultants. So, both students and teachers or professors need to learn how they are supposed to teach, or for students how we are supposed to learn.

2021_Participant 2

Students had *different experiences in interaction with classmates and teachers between online and campus education.* Several of them noted that *it was easier to communicate with each other* during the transition from online to on-campus learning given their previous digital interaction through lectures and the WhatsApp group, although some students found it *strange and unusual to meet their classmates* in person for the first time. However, the students experienced limited interaction with teachers within the program. They found it more challenging to engage with teachers compared to their classmates:

I think for me I can kind of remember that. Since we first started digitally and then we changed to the campus, we knew each other’s faces and how we interacted. So, in a way, it was kind of easier to change...After you’ve trained a little bit in the digital parts, then it gives you more confidence to talk in person. On the other side maybe, I was more shy to talk to the teachers...it was harder. I felt more the distance in a way, so it’s kind of the different sides, but maybe I felt nearer to my colleagues...

2020_Participant 2

I would say I mean. The first day when we met. It was, it was very weird to meet our colleagues. I mean, I’m not used to seeing them in real life. Some were shorter, some were taller, and you know it was a weird experience for the first day, I would say...

2020_Participant 7

### Students’ Well-Being and Engagement in Learning

**Figure 4:**
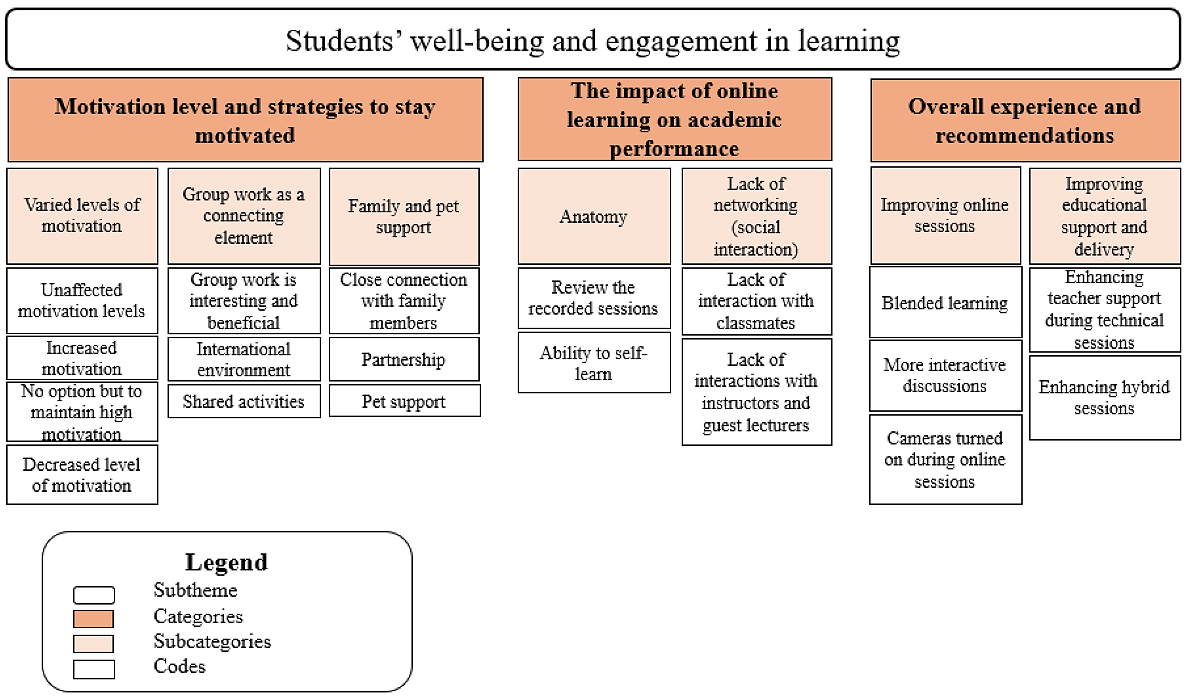
A hierarchical structure displaying the subtheme and codes for theme 3—students’ well-being and engagement in learning.

Figure 4 illustrates the relationship among the codes, subcategories, and categories for the subtheme *students’ well-being and engagement in learning.*

#### Motivation Level and Strategies to Stay Motivated

The *motivation* *levels* of students varied depending on their individual circumstances and also throughout the pandemic. Some students noted that their *motivation levels remained unaffected*, maintaining the same commitment to learning despite the pandemic. However, they expressed an *increased motivation* to meet people in person. Several students found that their motivation improved during the pandemic as they had more free time to plan upcoming courses, learn new skills, and engage in activities beyond their studies. Other students expressed that they had *no option but to maintain high motivation* due to visa requirements, family responsibilities, and future career plans. They highlighted that their relocation to Sweden was driven by the pursuit of a better future:

I would definitely say that when we moved to the campus, I felt more motivated not because of the learning, maybe because the motivation might be exactly the same, but also more, more motivated to meet people, to actually have an opportunity to meet different people because some of you have lived in Sweden and it’s hard most of the time because of the difference in the cultural aspects. So, I think that’s something that I remember being nice that I was happy to go to the campus and see these people and talk and learn with them.

2020_Participant 2

I think we as international students are here because you have your student visa, you have to study, and you have to pass to have your visa renewed. So, we don’t have this luxury...you know, I’m bored...I don’t want to study; I will skip it. No. We have to do this...We came here to study this program, and you have your plans, and you need to work to achieve your plans.

2019_Participant 2

...It’s the same motivation because I brought my kids [to Sweden] and I have to make the future. That is the motivation.

2019_Participant 3

Nevertheless, some students reported a *decreased level of motivation*. Some related this loss of motivation to their backgrounds, particularly feeling uncomfortable with certain courses such as programming and machine learning. Others mentioned that the darkness negatively affected their mood, although it did not impact their motivation to study:

If I talk about the motivation, yes it was reduced because I come from a medical background, and the technical things, especially the courses like programming and machine learning...There were so many new things that sometimes I felt I was lost...Within different things, all alone, and I don’t know if I’m the only one feeling like this or [if] there’s somebody else, or I mean, what else do I need to make it work better? But then I didn’t want to stop doing this...Because my main motivation was to learn technology and I wanted to complete it so that I know...

2019_Participant 1

I think the majority of the time that we had online lectures was during the darkest period in Sweden. And I think that affected the mood also because you then sat in front of your computer and it was dark, and then you sat inside the whole day pretty much. And then it was dark again. And so obviously that has an impact too, that it just happened to be during the most like the darkest period.

2021_Participant 4

Some students preferred online lectures due to the time and energy saved from morning routines such as waking up early, having breakfast, and preparing lunch. However, other students believed that lectures and interactions in person gave them more energy:

I really like remote learning and additionally, I’d like to add as I was saying in the beginning, I was able to be in another country because at that time my parents were living in another country and then me and [participant] actually could collaborate because we were at the same time zone.

2021_Participant 2

I will actually agree here with [participant] that also the lectures in person and interactions in person actually gave me more energy than they took, due to the fact that I was way more active. For example, I cycled to university. Then uh each morning and after class. Which always gives me an energy boost when I move more than also like just walking together on the campus, going for lunch, or getting a coffee or whatsoever. That also gave me I would stay way more energy than it took from me compared to when I would just be in my apartment. Pick up a coffee and then sit back again at the table...

2020_Participant 1

*Group work* was identified as a key factor for gaining and maintaining motivation during the pandemic. Many students believed that *engaging in group work was interesting and beneficial* throughout the program. The *international environment* played a crucial role in keeping students motivated. In addition, maintaining positive relationships with classmates and enjoying *shared activities* helped students stay motivated during the COVID-19 pandemic:

I would say that the group work made me more motivated. If I felt down. Honestly, we had very nice groups that we worked with. I enjoyed working with them and I learned a lot from them. But yeah, when you feel down and then we get a challenge to work on some project or some research or yeah, it was studying having some fun, and some jokes here and there. So, it wasn’*t* 100% serious mode. So yeah, group work affected...

2020_Participant 7

If it was just entirely remote, and I never had the opportunity to meet my classmates one-on-one, I don’t think I would be this motivated. Meeting them for the first time, seeing their various background, a lot of experience, and being able to tap into their own personal experiences and professional experiences gave me at least a lot of motivation, and a lot of interest in how better I can be...Or if it was just purely digital, or that involved me not traveling, I don’t think I would have been this motivated.

2021_Participant 3

Several students noted that having close connections with their *family, a partner, or a pet* played a significant role in keeping them motivated during the pandemic:

Yeah, I agree. Actually, that happened to me too with my husband. He was more of a body shadowing. Like, if someone else is working on your side like you, I don’t know. You get more folks. Somehow with cats, it’s different though. It may not be a routine or like force it to go out. To walk the cat...So, it would actually force me to get up, play a little bit with the cat, and then come back and it was really good.

2020_Participant 8

#### The Impact of Online Learning on Academic Performance

While some students reported no change in their academic performance, others observed an improvement in their overall achievement, among other reasons due to having the opportunity to *review the recorded sessions*. The students expressed a sense of adaptability to online learning, emphasizing their *ability to self-learn*. However, the students found it challenging to grasp technical aspects solely through online learning:

For me, I think that the pandemic situation increased my performance. Because lectures were recorded and then I used them again and again until I understood them. And then especially for the technical part...because we are not familiar with technology, then I used again and again for all those recorded things to understand...that was very effective for me.

2019_Participant 3

Students experienced a *lack of interaction with classmates, instructors, and guest lecturers*, hindering the exchange of ideas and discussion of questions. This lack of networking could potentially lead to delayed job opportunities:

...But yes, I agree with my colleagues that some from the social side there was a lack. Having a bigger network of friends or socializing with your friends and visiting companies in person, those who were giving us lectures like different healthcare companies were giving us guest lectures, but we couldn’t go in person to the companies to visit them...Because it was a pandemic, there were no, not many opportunities for summer jobs and internships, but not much open during that time. So, it was kind of that gap, which was present during [the pandemic]...

2020_Participant 6

#### Overall Experience and Recommendations

The students noted that the program altered their approach to problem-solving. However, they expressed a preference for *blended learning* over online learning alone as the latter had a negative impact on some, leading to feelings of isolation. In addition, the students provided suggestions for improvement in both online and campus-based sessions. They desired *more interactive discussions*, especially for technical sessions, to strengthen their knowledge after online lectures. Students also believed that having *cameras turned on during online sessions* would enhance the learning experience:

...So overall, it was a positive experience, in my opinion. I would say I learned a new way of learning I wasn’t used to, but sort of adaptability I would say. In terms of challenges, I would say I would agree with my colleagues about the social part. We were not able to socialize as compared to the normal or the offline study. It was very important. As well as building relationships with the instructors, teachers, and companies. And we were not able to do that. One other disadvantage was the interactive discussion with colleagues...

2020_Participant 7

...I can mention that not having the cameras on for example is another aspect that didn’t help in concentration, also being able to socialize with people because this is a very international program, and would be amazing just to be able to have been in the classroom from the beginning to the end...

2020_Participant 2

We got to see our peers in person and kind of create a connection and sit and eat lunch with each other and talk about things to get to know each other more, which actually can not only motivate us but also affect us in our ways of learning because we can get another perspective on a certain topic or something that we wouldn’t have gotten without talking to them during an informal event such as lunch, for instance...Again, I think a mix, a mix of online and on-site learning has made me at least reach my performance goals.

2021_Participant 2

The importance of having *teacher support* during the practical sessions in technical courses in the master’s program during the pandemic highlights a key factor in improving educational support in online learning:

Also, we have some practical sessions for our program, so we must have someone with us in the room to ask because there are technical things in programming. I don’t know where the error is in what I am doing, so I need a next eye to see what I’m doing.

2019_Participant 2

Enhancing hybrid sessions was mentioned as an area for improvement as students encountered numerous challenges during these sessions throughout the pandemic:

Yeah, I missed something. I just remembered that when we switched to in-person, there were some meetings hybrids. So people joined online while we were in the class and this was not really well organized or I don’t know if it was our problem or if it could be better, but I think it’s something that can be improved or just not having hybrid meetings, but I mean, I think we’re not prepared and we just had like 1 computer with the meeting, so only the teacher could hear the student who was at home.

2020_Participant 5

## Discussion

### Principal Findings

This paper reports the experience of master’s program students at a higher education institution due to the COVID-19 pandemic requiring adaptation to hybrid learning. Students experienced both benefits and challenges in relation to this. The transition between on-campus and online learning resulted in a hybrid learning era, which is likely here to stay. The students in this study appreciated the flexibility and autonomy provided by online learning, enabling them to integrate their studies into their daily lives. However, the rapid shift to online learning caused significant challenges, such as changes in social interactions, technical difficulties, and feelings of obligation rather than choice in adopting e-learning. The transition from on-campus to online learning caused a psychological impact on students and highlighted the need for better support and technological readiness to adapt to different learning environments. Returning to campus presented mixed experiences, with some students struggling to re-engage in face-to-face learning, whereas others faced challenges in adjusting to renewed forms of social and academic interactions. Students’ motivation varied during the pandemic. Group work, family support, and having pets played crucial roles in maintaining students’ moods. Despite the challenges, students were positive about hybrid learning to enhance future experiences, emphasizing the need for better networking opportunities and innovative strategies.

The findings of this study highlight implications for educators, students, and higher education institutions to embrace adaptation and foster innovation. These implications include the need for educators to stay current with the latest educational technologies and design teaching strategies and pedagogical approaches suited to both online and in-person settings to effectively foster student engagement. Students must be informed about the technological requirements for online learning and adequately prepared to meet them. Institutions play a critical role in ensuring equitable access to technology, guiding and supporting educators in adopting innovative tools and methods, and offering mental health resources to assist students in overcoming the challenges of evolving educational environments.

### Comparison to the Literature

Studies have previously reported both positive and negative consequences of the shift in learning environments. Naciri et al [10] have suggested that students generally responded positively to the rapid shift to online health science education during this crisis, expressing views on aspects such as acceptance, motivation, and engagement. Despite varying socioeconomic conditions across countries, certain key factors such as access to technology, basic computer literacy, well-designed online course pedagogy, and flexibility in learning consistently supported online education. However, students encountered challenges such as inconsistent internet access, difficulties with educational platforms, and hurdles in acquiring clinical skills online. These insights are crucial for enhancing the integration of these technologies into educational frameworks [10]. This is congruent with our results, where overall the students responded well to the rapid shift to online learning but reported both positive and negative outcomes, with challenges especially in computer laboratory sessions. Our findings also confirm the results of other studies [9,11] on the difficulties regarding limited internet access, technical problems, challenges related to adjustments from traditional to online formats, and impact on students’ well-being.

Students in our study were not entirely satisfied with the practical sessions in technical courses, highlighting the need for access to teacher assistance during these sessions. This contrasts with a study that reported satisfaction with clinical teaching and practical sessions remaining adequately high during the shift to e-learning. They also reported notable improvement in student satisfaction concerning course structure, instructor expertise, learning materials, and overall contentment with the courses, as well as a tendency for student grades to improve in the online format [8].

A review study [20] revealed that motivation and self-regulated learning were significant challenges, impacting students’ ability to engage critically with the material. It also showed varied attitudes toward online learning, with decreased satisfaction and emotional well-being in many students due to feelings of isolation and increased stress. These findings align with our results as students in this study also experienced loneliness and isolation, which led to a lack of focus on the studies.

Findings regarding the use of technology show that competence, perceived usefulness, ease of use, and facilitated implementation are predictors of learners’ attitudes toward and intentions to use technology [18,21,22]. Technologically capable students are likely to associate poor digital implementation by educators with lower satisfaction and self-efficacy. Integration of technology with a student-centered focus is likely to influence the development of autonomy and self-regulated learning [23,24]. For students enrolled in a health informatics program, where technology is a significant aspect of the learning curriculum, it could be extrapolated that they would likely be comfortable and be able to adapt to the use of technologies for learning. Therefore, the reported themes associated with technology use may be more significant for other groups of learners in different educational fields.

In addition to these findings, there has been a significant association reported between instructors’ use of effective teaching practices and student motivation. Alongside the quality of teaching and challenges associated with technology, prepandemic studies have indicated that motivation in online learning can be affected by demographic characteristics such as age, gender, employment status, income, and family obligations [22]. However, most studies were conducted at higher education institutions where students had a choice to enroll in online learning; learning in a hybrid environment may be different.

Online laboratory sessions in technical courses have shown some drawbacks in managing requests for help from student groups. During in-person sessions, teaching assistants can manage the requests of a group but, at the same time, give feedback to others (spontaneous interactions or questions that require a short answer). Using online learning tools with students divided into rooms, requests are managed only sequentially without allowing for spontaneous interactions. In addition, managing the request queue was more challenging for teaching assistants due to the unavailability of a specific function in the online learning tools. This seems to be in accordance with previous research on the topic [25-27].

### Implications for the Educators, Students, and Higher Education Institutions

The transition to online learning during the COVID-19 pandemic had significant implications for educators, students, and universities. Both educators and students had to make substantial adjustments in their teaching methods and learning styles, respectively. This study highlighted a greater emphasis on active learning strategies, self-discipline and time management, active participation in discussions and group work, and the development of flexibility and resilience.

Although our study focused on the experiences of students, the importance of educators in both the design and delivery of hybrid learning must be recognized. An online replication of a physical classroom can result in bored students and exhausted teachers, with both experiencing *Zoom fatigue*. A change in pedagogy is required to teach in the hybrid learning environment. Hybrid pedagogy is a development from blended learning using elements of both online and face-to-face learning, resulting in no separation between learners in the physical or digital space [13].

A significant issue related to hybrid learning is the required use of technologies. The health informatics students recognized both individual and organizational gaps in infrastructure and a lack of technological preparedness, which was experienced by many institutions. After the pandemic, it is critical that higher education institutions ensure that technology is leveraged to provide students with a good online, physical, or hybrid experience. The pandemic has highlighted the importance of preparedness and resilience in higher education for future crises. To support online and hybrid education, institutions need to invest in robust technological infrastructure and prioritize continuous professional development and training for educators. As highlighted in our study and similar research [9,10,28,29], there is a significant need to equip educators with the skills to adapt to the evolving educational landscape, particularly in the use of digital technologies. Educators need to develop skills to effectively use online platforms and tools to create engaging and interactive learning experiences. Common themes when designing quality online instruction that engages and motivates students are those of interaction, collaboration, communication, and discussion [21,30]. Online and hybrid learning require a greater focus on interactivity; this helps break the monotony and allows for student socialization. Therefore, engaging in ongoing professional development to stay updated with the latest educational technologies is a necessity for educators. Those students who had previously had the opportunity to meet in person on campus before transitioning to online learning may have had a closer sense of community than those whose educational experience transitioned in the opposite direction. Therefore, it is vital for instructors to build and foster a sense of community to keep students motivated. Overall, the onus is on educators to design and deliver quality teaching appropriate to the environment (web based or in person) to promote student engagement and encourage motivation to learn. To support these efforts, it is equally important that students are informed in advance of the requirements to attend classes online and be advised on the necessary technology (such as headphones, microphone, and camera). Furthermore, institutions should ensure access to technology by providing it on a short-term loan basis or guiding students in applying for grant funding, thereby ensuring equitable access to learning resources. Institutions should also guide and support educators to learn about and incorporate new technologies into their teaching practices [31-33]. In addition, providing extra resources and support to help students navigate the challenges of online learning is crucial. Given the impact that the shift had on students’ mental health, higher education institutions should provide mental health resources and support systems to assist students in managing these challenges. Students, educators, and institutions play a crucial role in informing policy makers regarding challenges and implications of future crises by sharing best practices for managing education emergencies. Providing feedback, sharing research findings, and offering concrete examples are essential for ensuring that policy makers are well informed and able to respond to the evolving needs of the educational community.

### Strengths and Limitations of This Study

This study is limited by its context-specific nature, focusing on a particular group of students within a specific educational environment (health informatics master’s students at KI), which may not fully capture the diverse experiences of learners in other regions, disciplines, or institutional settings. This may limit the generalizability of the results. However, the recommendations in the results can apply to other programs and institutions with similar educational systems and resources. The sample size in this study could be considered a limitation. However, saturation was achieved across the entire sample as no additional new themes emerged during the data analysis process. Although the number of participants from the 2019 cohort was low, the insights gathered from this group were consistent with those gathered from students from the other cohorts.

### Conclusions

The shift to hybrid learning in response to the COVID-19 pandemic presented both benefits and challenges for postgraduate students in higher education institutions. Technological preparedness, equitable access to technology, and educator training are crucial factors that institutions must address to support students’ learning experiences. In addition, fostering interaction, collaboration, communication, and discussion in online and hybrid learning environments is essential for engaging and motivating students. Ultimately, educators play a key role in designing and delivering quality teaching that promotes student engagement and encourages motivation to learn regardless of the learning environment.

Future studies should aim to explore the impact of the COVID-19 pandemic on students’ learning experiences across varied contexts, incorporating cross-institutional comparisons to develop a more comprehensive understanding of how different factors influence learning experiences and outcomes in hybrid and online education. In addition, it is of great importance that future studies examine new teaching methods and pedagogical approaches that can enhance students’ engagement and increase communication and interaction between students in hybrid education. Finally, it is crucial to develop strategies and policies that prepare higher education institutions for crises, ensuring that they can effectively transition between educational environments—whether shifting from in-person to online or hybrid modes—while maintaining educational continuity and quality. Future research could use case studies to investigate institutional responses and apply principles of action research to collaboratively develop and refine teaching strategies and crisis management policies.

## Abbreviations

COREQ: Consolidated Criteria for Reporting Qualitative Research
KI: Karolinska Institutet
